# “Deficits Don’t Matter”: Abundance, Indebtedness and American Culture

**DOI:** 10.1007/s12115-015-9879-1

**Published:** 2015-03-03

**Authors:** Rob Kroes

**Affiliations:** P.O. Box 19268, 1000 GG Amsterdam, The Netherlands

**Keywords:** U.S. economic policy, American character, U.S. culture, Cultural critique, Neo-liberalism

## Abstract

If deficits, nor defaults, don’t really matter anymore, what sign of our times is it? What has changed from the days that Franklin Delano Roosevelt risked the fragile economic recovery from the great depression by returning, in 1937, to the standard of his economic orthodoxy, a belief in fiscal rectitude and anaversion to debts and deficits? If that was a sign of a certain American character, what has happened to it? A massive shift in public culture must have occurred, affecting people’s views on public probity and political rectitude. The following is an attempt to trace some of the main shifts on the way to our present quandary.

In a conversation between vice-president Dick Cheney and Secretary of the Treasury Paul O’Neill Cheney is quoted as saying: “Reagan proved deficits don’t matter.” It is one telling quotation among many that show the prevailing hubris in government circles at the time, a belief of having cast off the shackles of economic reality. The hubris is still there, unabated. In a review of two books tracing back the lines of development of this hubris to the days of Nixon and Reagan, Robert G. Kaiser reminds his readers of the 2013 House of Representatives vote to raise the national debt ceiling. Failing to do so would effectively force the United States to default on its obligations to creditors. The ceiling was duly raised, but 144 Republican members said no. A number among them expressed confidence that *default wouldn’t really matter* (my italics). Kaiser goes on to say: “…that a 144 members of the House were willing to cast a vote to default on the full faith and credit of the United States is a sign of our times.”

If deficits, nor defaults, don’t really matter anymore, what sign of our times is it? What has changed from the days that Franklin Delano Roosevelt risked the fragile economic recovery from the great depression by returning, in 1937, to the standard of his economic orthodoxy, a belief in fiscal rectitude and an aversion to debts and deficits? If that was a sign of a certain American character, what has happened to it? A massive shift in public culture must have occurred, affecting people’s views on public probity and political rectitude. The following is an attempt to trace some of the main shifts on the way to our present quandary.^1^

## Debts in Abundance

In the early days of what its guiding lights and eager followers called the American Studies Movement, in the United States in the 1930s and ‘40s, and spreading abroad into the early Cold War years under U.S. cultural diplomacy auspices, the quest was on for establishing and defining what was variously called the American identity, the American character, the American mind, or even the American Self. Agreement was never reached, which only added to the appeal of the quest. Literary studies, the study of history, and the newly reputable social sciences were all yoked together in the hot pursuit of this elusive, if not chimeric, target. For good measure, rival stories of origin were thrown into the mix. Puritan origins were a strong contender, from Perry Miller’s *Errand into the Wilderness* to Sacvan Bercovitch’s *The Puritan Origins of the American Self*. But so were stories of America’s given natural resources, of America as cornucopia, as in David Potter’s *People of Plenty*, or stories of America as an ideological blank sheet, open to be inscribed with European liberalism, to the exclusion of its European rivals, as in Louis Hartz’s *The Liberal Tradition in America*. Others were working parallel veins, such as Richard Hofstadter, and Daniel Boorstin. They were all in their own way varying on the theme of American exceptionalism, exploring themes of “divine election” or “chosenness,” or of manifest destiny and the fore-ordained westward course of empire, or of geographical determinism, following in the footsteps of Frederick Jackson Turner’s frontier thesis. As for the historians among these 1950s’ writers, reference is sometimes made to them as constituting a “ school,” the school of the consensus historians. The word is felicitous, highlighting as it does a crucial pre-condition for the existence of something like an American Mind, or an American Self. It takes a shared history of like-mindedness, a national—if not notional—consensus, for there to be such a thing as one national identity, or one national character.

Yet, given this fevered quest for one shared national character, recognizably there in each of its individual carriers, it is nothing but utterly ironic that much of the intellectual debate in the United States in the 1950s was set by a book that was out to explore “ the changing American character.” I am referring of course to *The Lonely Crowd,* a book commonly linked to the name of Harvard sociologist David Riesman, but really the result of team work.^2^ Rather than bringing historical data together to buttress the case for one national identity, Riesman a.o. suggested that historically America may have known two or three modal characters, following each other in time, and each with its typical modes of behavior, cultural tastes and appetites, and individual character structure. I may remind you here of the two main character structures that Riesman recognized. Historically, he sees “inner-directed man” give way to “other-directed man.” Inner-directed man is the self-reliant and self-sufficient character, redolent of the Puritan individual guided by an inner sense of righteousness and direction, an inner compass, as Riesman metaphorically called it. Other-directed man is the successor personality type, entering the stage in the wake of radical social transformations.

In a new era of greater social interdependence and much more rapid social and cultural change, parents are no longer able to equip their children for life with their own inner compass. They now need to be trained to become social animals, taking their cues on a daily basis from their peers, adapting their behavior and tastes accordingly, adopting the hue and color of the settings they find themselves in. They now orient themselves by using, not an inner compass, but what Riesman calls their inner radar. Other classic texts from the 1950s further fleshed out this type, such as William H. Whyte’s *The Organization Man*, highlighting the structural setting in which increasing numbers of people spent their working lives, the bureaucratic setting of the large-scale business corporation or government organization.

Riesman, as I shall argue, is only one among many authors who set out to recognize tidal changes in dominant character types in American history as they relate to underlying social and economic changes. As people’s characteristic patterns of dependence—financial dependence through indebtedness critically among them—change and as they lose such measures of autonomy as they might have grown used to seeing as rightfully theirs, character structures and larger patterns of culture are assumed to reflect these changes and to turn into their symbolic representations.

Of course, as the world became increasingly bureaucratic in its patterns of organizing society and as people became enmeshed in large-scale structures controlling their lives, they no longer have the option, as Polonius, in Shakespeare’s *Hamlet* (ACT I SCENE III) puts it to his son Laertes: “This above all, to thine own self be true.” They have to play by the ever-changing rules of social games, that Riesman, for one, took sardonic pleasure in analyzing. But my point is, Riesman was not the only one to do this. He stands in a long line of social critics who read the signs of the times in the changing behavior patterns of their contemporaries. I will take you on a tour d’horizon of such critical writing. Can they teach us anything on the ways in which patterns of dependence—financial dependence included—have been reflected in the modes and tones of larger cultural eras. This will then lead to my ultimate question concerning the current state of affairs in America. What possible cultural reflections can we see of a current situation where all of America, at every level, internationally as a sovereign state, nationally as a government, and down from there to the level of individual businesses and families, is in deficit, on a scale of indebtedness unprecedented in its national history? Are there any clear signs of cultural characters emerging to reflect this state of affairs?

## Changes in Cultural Character

It would be tempting to see Riesman’s *The Lonely Crowd* as a nodal point, a conceptual hub, where several lines of intellectual gestation came together before it would inspire later portraits of American culture in broadly the same vein. Undoubtedly later work, like Christopher Lasch’s 1979 study of *The Culture of Narcissism: American Life in an Age of Diminishing Expectations*, can be seen to echo some of Riesman’s central characters, yet between the 1950s and the 1970s dramatic shifts had occurred in America’s structural setting. A postwar era of explosive growth and all its unsettling impact on the population’s rising expectations had, by the early 1970s, turned into its opposite, of economic stagflation and diminishing expectations of individual life chances. As Lasch puts it, every age develops its own peculiar forms of pathology, which express in exaggerated form its underlying character structure. The pathology that Lasch chose to use as the metaphor for the prevailing character structure of the “Me-decade” is narcissism. It is an age that had seen the eclipse of individual achievement and of the satisfactions of its pursuit. “Today men seek the kind of approval that applauds not their actions but their personal attributes. They wish to be not so much esteemed as admired.” (p.59)

Lasch stands in a long line of critics of mass society. He located the pivot of modern psychic development in the rise of mass production, with its concomitant deskilling of workers, destruction of economic independence, change in relations of authority from personal to abstract, and the professionalization of education, management, mental health, social welfare, and the like. The result of those epochal changes was a drastic change in the socialization of children. *Individuation*—the process of the formation of individual selves—largely consists of the gradual reduction in scale of infantile fantasies of omnipotence and helplessness, accompanied by the child’s modest but growing sense of mastery, continually measured against its human and material surroundings. Formerly, the presence of potent but fallible individuals, economically self-sufficient, with final legal and moral authority over their children’s upbringing, provided one kind of template for the growing child’s psychic development. As fathers (and increasingly mothers) become employees, with the family’s economic survival dependent on remote, abstract corporate authorities, and as caretaking parents were increasingly supervised or replaced by educational, medical, and social-welfare bureaucracies, the template changed. The child now has no human-size authority figures in the immediate environment against which to measure itself and so reduce its fantasies to human scale. As a result, it continues to alternate between fantasies of omnipotence and helplessness. This makes acceptance of limits, finitude, and death more difficult, which in turn makes commitment and perseverance of any kind—civic, artistic, sexual, parental—more difficult.

The result is narcissism, which Lasch, in the opening pages of *Culture of Narcissism*, described thus:

Having surrendered most of his technical skills to the corporation, [the contemporary American] can no longer provide for his material needs. As the family loses not only its productive functions but many of its reproductive functions as well, men and women no longer manage even to raise their children without the help of certified experts. The atrophy of older traditions of self-help has eroded everyday competence, in one area after another, and has made the individual dependent on the state, the corporation, and other bureaucracies.

Narcissism represents the psychological dimension of this dependence. Notwithstanding his occasional illusions of omnipotence, the narcissist depends on others to validate his self-esteem. He cannot live without an admiring audience. His apparent freedom from family ties and institutional constraints does not free him to stand alone or to glory in his individuality. On the contrary, it contributes to his insecurity, which he can overcome only by seeing his “grandiose self” reflected in the attentions of others, or by attaching himself to those who radiate celebrity, power, and charisma. For the narcissist, the world is a mirror, whereas the rugged individualist saw it as an empty wilderness to be shaped to his own design. Narcissism refers to a weak, ungrounded, defensive, insecure, manipulative self—what Lasch’s next book, eponymously titled, labeled “the minimal self.”

Yet readers may be forgiven if they recognize in Lasch’s narcissistic personality the traits of Riesman’s other-directed man. Lasch vehemently denies the similarity, the family likeness. As he argues, “Americans have not really become more sociable and cooperative, as the theorists of other-direction and conformity would like us to believe, they have merely become more adept at exploiting the conventions of interpersonal relations for their own benefit.” (p. 66) This could only be argued by someone totally missing out on the sardonic pleasure Riesman takes in analyzing precisely the one-upmanship involved in the interactions of other-directed persons, with their eye on the main chance to upstage others. Riesman’s other-directed man is more than just the incarnation of Dale Carnegie’s smooth social operator, the central character of his immensely successful 1936 “ How to…” book, and held up as a model for all to follow on their way to success, “winning friends and influencing people.” Carnegie did catch unfailingly a cultural shift underway ever since the 1920s, a demotion of certain long-respected virtues, where character gave way to personality, self-control to self-fulfillment, industry and thrift to skill at handling people. Carnegie’s engineering of the self constructed a model of modern individualism composed entirely of serial images, disjointed, lacking any logic of inner cohesion, with no sturdy commitments or beliefs, no firm moral standards, *no authentic and rooted core of self,* (words that might have been Lasch’s, but are not).^3^ In Carnegie’s view, it consisted only of a pliable personality eager to please others and advance socially and economically.

All this we may recognize in Riesman’s type of the other-directed man, or for that matter—think of “no authentic and rooted core of self”—in Lasch’s narcissist. But there is so much more that feeds into Riesman’s perspective, and into his tongue-in-cheek, picaresque pantheon of tricksters and confidence men. After all, who can forget the unforgettable personae that Riesman conjured up, like the inside-dopester ( a word it took me years to probe in its depths of American colloquial resonance)? There are echoes here of the Chicago School in Sociology, and central figures like George Herbert Mead and Herbert George Blumer and their ideas on symbolic interactionism, echoes also of seminal insights into the social construction of the self, as a process of ongoing social negotiations and interactions like so many feedback loops informing people’s trajectory towards self-definition. One is also reminded of Erving Goffman, another Chicago School name and author of the classic *The Presentation of Self in Everyday Life*. They are all examples of a special intellectual sensibility and an alertness to concepts like personality and culture seen as essentially open and in flux. Goffman in particular had an ear and an eye for the trickster element in all this, for the histrionics and theatricality in people’s social strategies.

Yet another resonance that we may pick up reading Riesman is the unmistakable voice of Thorstein Veblen, odd man out in the history of American sociology and economics, yet a one-man fount of insight, critique and sardonic wit. He wrote at a time, in the late 19th, early 20th century, of rapid transformation across a wide swathe of life in America. Relative latecomer to industrialization and urbanization that America was, much like Germany in Europe in the fevered catch-up of its so-called “Gründerjahre”—the years of industrial take-off—students of society in both countries invented new concepts for analytically capturing the advent of modernization. These were the years that Alan Trachtenberg would call the age of incorporation, the years in which a business paradigm of large-scale rational organization began to dictate most people’s workaday lives. Not only had the systems of production dramatically increased in scale, so had the attending systems of control and governance. Increasing numbers of people had become enmeshed in a web of bureaucracy, putting them at an ever growing remove from the actual line of production. A parallel world arose, of staff workers alongside line workers, a world of growing abstractness, losing point and purpose for those involved. This new world was explored and analysed in Germany by leading early sociologists like Max Weber or Alfred Tönnies. Weber came up with the metaphor of the “iron cage” to capture the social experience of life in a bureaucratic setting. Tönnies introduced the pair of opposed concepts of *Gemeinschaft* versus *Gesellschaft*, words that in their English translation lose the evocative force they have in German. Early American sociology came up with a felicitous parallel, though, opposing primary to secondary social relations.

In this view the rich affective resonance of primary groups, like the family, neighborhood and local community, stood opposed to the cold and formal qualities of secondary relations, connecting people merely through formalized social roles. The latter evoke the world of the office window, bank tellers, secretaries and desk workers, a world that was increasingly liquid, losing form and meaning for the self-definition of all those involved, eating away at the many-stranded bonds of civil society, eroding its social capital. In this “Great Transformation,” as Karl Polanyi memorably called it, a self-regulating market was to emerge, turning human beings and the natural environment into commodities.^4^

Yet, as many observers at the time noted, human beings did not take this lying down. New social stages for public self-definition evolved which allowed people to explore early forms of a consumption culture with a view to setting themselves apart from others and distinguish themselves in the public eye. This is the stage that Veblen exposed in his first published book, *The Theory of the Leisure Class*. In it he lets his eyes roam across the wide array of strategies of social distinction through the ostentation of spending behavior. His sardonic wit coined phrases for the description of this behavior that survive until the present day, words such as “conspicuous consumption,” “invidious distinction,” or “marginal differentiation.” 5 The latter term in particular survived through Freud’s reflections on the narcissism of minor differences for the exalted display of individuality. In current post-modern analyses, the strategic point in using this form of narcissism is to achieve a superficial sense of one’s own uniqueness, an *ersatz* sense of individual distinctness which is only a mask for an underlying uniformity and sameness. If Veblen is to rank as a social and cultural critic here is the reason why: he exposed the underlying vacuity of an era whose cultural parameters were set by the robber baron and the alienated office worker. If there is a dialectic at work here, it is that between the alienated many and the extortionist few who manage to get something for nothing. It is the group who, not unlike Karl Marx’s expropriating capitalists, have kept their eyes on the main chance and the main prize. With characteristic sarcasm Veblen calls them the impropriators, reviving an old word from the world of canonic law to highlight the impropriety of expropriation. “So there has been incorporated in American commonsense and has grown into American practice the presumption that all the natural resources of the country must of right be held in private ownership , by those persons who have been lucky enough or shrewd enough to take them over according to the rules in such cases made and provided, or by those who have acquired title from these original impropriators.”^6^

As one further interpretative revisit of the era reminds us, the telling metaphor for the period may be its fashionable middle-class affliction which went by the name of neurasthenia, best described as the physical symptoms of French poet Paul Verlaine’s “langueur monotone.” Neurasthenia, as author T.J. Jackson Lears suggests in his *No Place of Grace*,^7^ was the medicalized expression, if not representation, of a more general feeling that in view of modern life having grown dry and passionless, one must somehow try to regenerate a lost intensity of feeling. But not only that. As Jackson Lears points out: “Late Victorians felt hemmed in by busyness, clutter, propriety; they were beset by religious anxieties, and by debilitating worries about financial insecurity.” There was a financial dimension to the way Americans responded to the transformation of their collective life in the late 19th century. It is what drove the new games played with the commodities produced by America’s industrial machine, transforming them into signs and symbols of material success in a social arena shot through with status anxieties and feelings of economic insecurity. Whether or not individual Americans came out on top, they were all equally drawn into a new social game that before long would form an integral part of America’s nascent culture of consumption.

That cultural transformation came with its own key word, abundance. At long last the American Dream could appear to have come into its own, unlocking a veritable cornucopia, fulfilling what had in fact been age-old European fairytale dreams of a land of plenty, “un pays de Cocaigne” (which today does not sound right if translated back as a land of cocaine) or for that matter a Marxian dream of a realm of scarcity being replaced by a realm of affluence. Entering the 1920s America seemed to have led the way into this realm, even in the eyes of assorted European socialists, syndicalists and even communists.

Jackson Lears made abundance the topic of a separate study, published as *Fables of Abundance: A Cultural History of Advertising in America*.^8^ Similarly, one of the seminal authors in this field, father of contemporary cultural history and cultural studies as we know them today, Warren Susman, suggested as the backdrop for his explorations of 20th-century cultural trends in America the single word abundance.^9^ “…struggling to articulate for myself and my students some definition of what our culture is like and how it got this way, I find that I was developing almost unconsciously a way of understanding American culture: I was coming to see America through the notion of ‘the culture of abundance.’” (p. xx) As he came to see it, one of the fundamental conflicts of twentieth-century America is between two cultures—an older culture, often loosely labeled Puritan-republican, producer-capitalist culture, and a newly emerging culture of abundance. As those familiar with his work will remember, Susman really made his mark developing approaches to the problem of capturing signs of this cultural transformation taking place. Working in a pre-digital age, he truly morphed into a one-man logarithm, pioneering work that would later be known as data-mining, producing word clouds as if he were a cutting-edge digital historian. Word clouds? Yes, world clouds. With characteristic inquisitiveness and sensitivity to the uses of language he struck upon submerged shifts in the frequency with which words were used, unearthing words that were becoming the shibboleths of their age. Words came in packages, cohering through their contextual uses; some were on the way out, falling into disuse, others pushed forward. And Susman presented them as word clouds. Here is Susman at work: “Initial investigations to answer such questions yielded suggestions of significant transformation. Key words began to show themselves: *plenty, play, leisure, recreation, self-fulfillment, dreams, pleasure, immediate gratification, personality, public relations, publicity, celebrity.* Everywhere there was a new emphasis on buying, spending, and consuming.” (p. xxiv) In a brilliant chapter he shows how the older culture, Puritan-republican, producer-capitalist demanded something it called “character,” which stressed moral qualities, deeply ingrained, whereas the newer culture insisted on “personality,” which emphasized being liked and admired. It is not hard to see these two key words as foreshadowing Riesman’s later social types of the inner-directed man and the other-directed man, only taken forward in time to the turn of the 19th century.

Susman and Jackson Lears both mention advertising as a critical new use of new technologies of mass communication for the new world of abundance and mass consumption to function smoothly. Susman even mentions one of advertising’s central functions lying in its actively *creating wants*, *inducing* consumer demand for novel products entering the market. Advertising in that sense plays a critical role in balancing supply and demand, in channeling production to meet consumption. And in fact, one of the standard accounts of the causes of the Great Depression is precisely in terms of over-production, of a failure of market mechanisms. But there is such a thing as under-consumption, of lagging demand due to stagnant purchasing power among the mass of consumers. And the remarkable thing, going over Susman’s word clouds as they hang about the capitalized word ABUNDANCE, is the total absence of words connected to debt, insolvency and poverty.

There is one student of the American Dream of Abundance who has his eye out for this different set of words, which, if they form a cloud, it is surely a storm cloud. Roland Marchand, in his *Advertising the American Dream: Making Way For Modernity, 1920–1940*, in fact makes this central point that the advent of consumer culture brought with it a radical break with older virtues such as frugality, financial prudence and a general aversion to debt. All this went overboard in the 1920s. A general buy-now, pay-later attitude was advertised in its own right as the thoroughly modern way to go. As Lizabeth Cohen reminds us, all expenditure for private consumption came to be seen in the later 1930s and ‘40s as good citizenship, keeping the national economy going and growing.^10^ But much of the spending critically hinged on financing mechanisms, through installment plans, charge cards, and other forms of deficit financing, and let individual consumers blithely run up private debts. Yet never did the debts collectively amassed in this 1920s’ trial run of consumerism reach the heights they would a half century later. Nor did they set a tone of cultural life or produce a new social type as they may have much later. Christopher Lasch may have been on to something when he set out to explore a novel social character structure in his *Age of Narcissism*, or for that matter in his *Haven in a Heartless World*, against the background of what he termed an “age of diminishing expectations.”

## America’s Cultural Character at the End of Empire

Given the immense debt overhang at every aggregate level of American society, how does this situation reflect in the writing of social commentators, historians and cultural critics? What forms of representation, what symbolic reflections, can we recognize? What sort of Colossus is America today, sole remaining superpower, a hegemon by any measure, yet deeply indebted to the main rival to its power, China? Are what we are witnessing the signs of the end of empire, of its unstoppable decline?

In one analysis of America’s status as an empire, Charles Maier makes the following interesting distinction. Asking himself the question whether America can rank as an empire among empires, and if so on what grounds compared to earlier historical cases, he distinguishes two historical stages in the American case: America as an empire of production followed by America as an empire of consumption. By the latter term Maier does not, as one may briefly expect, refer to America’s era of consumerism and the cultural forms attending it. What he evokes is not America as an empire with the full panoply of the soft power of its culture of consumerism. No, he wishes to bring out the stark contrast between America as the marvel of productive prowess that it was in the mid-20th century and the America that can no longer produce all it wishes to consume. So from being an net exporter of goods it produced, it turned into a net importer, with its trade balance duly reflecting this shift. From being a creditor nation it had turned into a debtor nation, losing independence and freedom of action in the process. Now if there are signs of empire declining to be read in these secular trends, of an empire depending not only on borrowed money, but on borrowed time, are they beginning to dawn on the broader American population? And if so, what effect do they have on America’s over-all state of mind?

It doesn’t take the documentary eye of a Michael Moore to conjure up a visual America replete with the signs of decay, decadence and defeat. Forgotten veterans of America’s far-away wars—far-away geographically, but more dramatically far-away from the public consciousness, repressed and pushed out of the public sphere—bring to mind Georg Gross’ depictions of World War I veterans limping through Berlin streets. There is a seething anger among Americans, aimed at the impotence of presidents, of politics, aimed at the one-percent of the obscenely rich, an anger thrashing about wildly, yet unable to find meaningful expression, other than in a politics of resentment, Tea party politics, gun-toting and empty patriotic gestures. It is the anger of the self-styled militia, vindictive and utterly nihilistic. If there is a changing American character to be recognized here, we need a Richard Hofstadter to do it for us. After all, he has done it before, magisterially describing for us the paranoid style in American politics.^11^

And yet, paranoia as a metaphor seems to cover only part of what I wish to capture. Paranoia does not stretch any farther than the lunatic fringe whose conspiratorial fantasies see the federal government in Washington, DC. as one big plot against the freedoms of individual Americans. As a metaphor it does not begin to account for statistics that show the proportion of Americans who still trust a government institution like Congress to be a meager 7 % or the proportion of Americans who expect their children to be worse off than they are to be a staggering two thirds. These are signs of collective disaffection in the face of a dysfunctional political system and of a collective sense of loss of control and direction. Nor is it only a matter of politics and a lack of citizen empowerment. It doesn’t take the conspiratorial view of Hofstadter’s paranoid style to see the economic system as producing ever growing income and property gaps. You don’t have to be a Riesman-like inside-dopester to take seriously a view of the world of finance as driven by self-interest, geared against standards of decency and public service, a view presented in an award-winning, muck-raking documentary film like *Inside Job*.^12^ It is a world of sharks, sharpers, and conmen, where banks are betting against their own customers, and where suckers are born every minute. If all this has led to a massive breakdown of social trust, it is not so much a sign of paranoia as it is of rational people who duly feel duped.

There is a number of best-selling books that have all tried to diagnose this mounting distrust, this erosion of America’s social capital or of its habits of the heart, all noticing secular trends away from golden ages of civic enthusiasm and levels of engaged public debate and of trust worthy of a republic.^13^ They all notice a secular slippage away from Tocquevillean standards of a multi-stranded associative life, of an erosion of civil religion and civic participation, of a loss of social capital. They all see the downward slope of democratic vigor, yet tend to miss the aspect of a rational assessment of reality behind it. Rather than people bowling alone because they no longer join social clubs, people have chosen to withdraw from politics, have withdrawn their trust from economic institutions, and no longer believe what they are told by talking heads on their TV’s. They have done this because they have knowledge of Wall Street inside jobs and related fraud, not because they have let themselves be passively “framed” by the relentless distortion of public debate that now passes for TV journalism. Outside the dysfunctional media landscape, where enlightened public debate has been bought out by private capital and the nihilistic ideology of corporate interests, many are now exploring ways to restore “social capital,” finding ways of discussing a political agenda that no longer will get a fair hearing in the traditional halls of the republic. If the American character is morphing once again, it is not in the direction of people bowling alone, but toward the “ agora,” the online marketplace of ideas and organized action, of life in cyberspace. It may not be the only crowd roaming America’s public space, a lonely crowd it certainly isn’t.

This ironically takes us back to the theme of “primary groups” as the mainstay of Tocqueville’s civil society. Ever since Polanyi’s “Great Transformation,” or Trachtenberg’s “incorporation” of America, there has been an ongoing quest for signs of primary groups surviving and kicking. If the advent of modernity meant the demise of communitarian settings and primary relationships, students of society kept spotting primary groups in the most unlikely settings. In urban life, where the early Chicago School had explored “urbanism as a way of life,” and celebrated its modernity, individualism, and cosmopolitanism, integrated community structures were found to have survived, even thrived, as Herbert Gans showed in his *Urban Villagers*. If the advent of new media, such as radio, spawned big national broadcasting corporations, this need not have been the only, pre-ordained outcome. As Lizabeth Cohen showed in her *Making a New Deal*, working-class communities in a metropolis like Chicago for a brief period managed to harness the medium to give voice to the local community rather than the impersonal corporatism that characterizes the current media landscape. If, in the world of industrial work, Taylorism and the rationalization of production meant the reduction of individual workers to mere cogs in a machine, early industrial relations research in, e.g., Elton Mayo’s classic Hawthorne studies pointed up the power of informal groups on the work floor to bend the rigidity of imposed production norms. If in politics the individual voter was seen as increasingly alienated and atomized, studies at the local level once again showed the role played by informal groups, inspiring an interpretive paradigm, popular in the 1950s, known as pluralist elitism. Robert Dahl’s *Who Governs* is the classic reference here, although David Riesman’s *Lonely Crowd* memorably contributed to the new paradigm with its view of what he called “veto groups,” informal groups strong enough to block political decisions they do not like, yet insufficiently strong to have things their own way. It is basically a return to classic Tocquevillean intimations about American politics as the interplay of a multiplicity of groups.^14^

Yet, undeniably, all these examples can be seen as so many exercises in nostalgia, as studies of lost causes. If processes of incorporation, under auspices of an impersonal neo-liberalism, have now gone global, can we possibly conceive of a response along “primary group” lines to get us out of the “iron cage” of globalization? For an answer we might look at the ways in which an international commonwealth, literally a republic, a *res publica*, organizes itself around issues of human rights, the environment, and economic inequality, through the network possibilities of the World Wide Web. In areas like these, on a global scale, the social capital is being formed of a civil society that is truly trans-national.

## Footnotes

1 Ron Suskind, *The Price of Loyalty: George W. Bush, the White House, and the Education of Paul O’Neill* (New York: Simon and Schuster, 2004) 334. Robert G. Kaiser, “Our Conservative, Criminal, Politicians,” *The New York Review of Books*, (November 6, 2014) 56.

2 David Riesman, Nathan Glazer, Reuel Denney, *The Lonely Crowd: A Study of the Changing American Character* (New haven: Yale University Press, 1950)

3 The words are quoted from Steven Watts, *Self-Help Messiah: Dale Carnegie and Success in Modern America* (Other Press, 2014)

4 Karl Polanyi, *The Great Transformation* [Foreword by Robert M. MacIver] ( New York: Farrar & Rinehart., 1944)

5 S. Freud, *Civilization and Its Discontents* (New York: W.W. Norton & Company, Inc. , 1961)

6 Wesley C. Mitchell, ed., *What Veblen Taught: Selections from the Writings of Thorstein Veblen* (New York: The Viking Press, 1945) 372

7 T.J. Jackson Lears, *No Place of Grace: Anti-Modernism and the Transformation of American Culture, 1880–1920* (Chicago: University of Chicago Press, 1981)

8 T.J. Jackson Lears, *Fables of Abundance: A Cultural History of Advertising in America* (New York: Basic books, 1994)

9 Warren I. Susman, *Culture As History: The Transformation of American Society in the Twentieth Century* (New York: Pantheon Books, 1973)

10 L. Cohen, *A Consumers’ Republic: The Politics of Mass Consumption in Postwar America* (New York: Alfred A. Knopf, 2003) Ch. 3.

11 Richard Hofstadter, *The Paranoid Style in American Politics and other essays* (Cambridge, Mass.: Harvard University Press, 1952)

12 Charles Ferguson, *Inside Job* (Sony Pictures, 2010)

13 Robert N. Bellah, a.o., *Habits of the Heart: Individualism and Commitment in American Life* (1985); Robert D. Putnam, *Bowling Alone: America’s Declining Social Capital* (New York: Simon and Schuster, 2000) Francis Fukuyama, *Trust: The Civil Virtues and the creation of Prosperity* (New York: The free Press, 1995)

14 Herbert J. Gans, *The Urban Villagers* (New York: Free Press of Glencoe, 1962); Lizabeth Cohen, *Making a New Deal: Industrial Workers in Chicago, 1919–1939* (New York: Cambridge University Press, 1990); F.J. Roethlisberger, William J. Dickson, *Management and the Worker* (Cambridge: Harvard University Press, 1939); Robert A. Dahl, *Who Governs? Democracy and Power in an American City* ( New Haven/London: Yale University Press, 1961)

